# Identification of factors affecting student academic burnout in online education during the COVID-19 pandemic using grey Delphi and grey-DEMATEL techniques

**DOI:** 10.1038/s41598-024-53233-7

**Published:** 2024-02-17

**Authors:** Andrea Aria, Parivash Jafari, Maryam Behifar

**Affiliations:** 1 CIS Department, Robinson College of Business, Georgia State University, 35 Broad St. NW, Atlanta, GA 30303 USA; 2 Department of Educational Administration, School of Management and Economics, Science and Research Branch, Islamic Azad University, Tehran, Iran; 3 Department of Technology Management, School of Management and Economics, Science and Research Branch, Islamic Azad University, Tehran, Iran

**Keywords:** Psychology, Human behaviour

## Abstract

The coronavirus outbreak caused most education institutions to shift to online education. One of the consequences of the pandemic and spread of online education was the prevalence of academic burnout among students has been students' academic burnout. Accordingly, it is necessary to identify the influential factors to reduce burnout. This study aimed to identify the factors affecting academic burnout and their cause-effect relationships. For this purpose, to conduct the gray Delphi technique, a questionnaire was administered to a randomly selected sample consisted of 86 graduate students of the Islamic Azad University, Science and Research Branch. In addition, a randomly selected sample of 37 graduate students filled a matrix questionnaire Collected data were analyzed using the Gray-DEMATEL technique. As a result of Gray Delphi screening, out of the 43 sub-factors identified, six sub-factors were eliminated and 37 sub-factors in 7 main factors were determined as factors affecting students' academic burnout. Analysis of the collected data using the Grey-DEMATEL technique revealed that among the seven main factors, Technology infrastructure, institutional facilities, and faculty characteristics are in the net cause category, respectively. The classroom environment and course structure, the social-emotional factor, the characteristics of students, and the home and family environment are in the net effect category. Technology infrastructure is the most influential factor that affects other factors. Identifying effective factors and the causal relationships between them have significant implications for policymakers and academic planners to prevent and reduce student burnout in online environment by focusing on the most influential factors.

## Introduction

Covid-19 pandemic affected all aspects of human life and surprised people and institutions in an unexpected way. The coronavirus outbreak in three years affected almost all aspects of human life. One of the global measures to protect the health and safety of most people against COVID-19 was social distancing, which led to the closure of educational centers and the shift to online education. Rapid and unplanned transition from face-to-face education to online education combined with concerns about coronavirus infection led to stressful learning environments and the risk of increasing burnout among students. Maslach and Jackson (1981) defined burnout as having three characteristics: emotional exhaustion, depersonalization or pessimism, and feelings of inadequacy and inefficacy^1^. Schaufeli, Martinez, Marques, Salanova, Bakker (2002) extended burnout to educational settings^2^, and Bresó, Salanova, Schaufeli (2007) likened students to employees who may be at risk of burnout, arguing that they, like staff, have to engage in structured activities such as class attendance, doing assignments and passing exams to achieve specific goals^3,4^. Student academic burnout has been defined as a psychological syndrome and a long-term response to chronic interpersonal stressors in a social context, which is characterized by exhaustion due to high academic demands and overload, feelings of cynicism and detachment from the studies and academic activities, and a feeling of academic inefficacy as a student^2,5^. Studies show that student academic burnout leads to many negative consequences such as physical problems and mental disorders, lower Quality of Life, decreased motivation^6^, low academic performance^2,6–8^, Decreased life satisfaction^9^, and less satisfaction with studies^10^, and intention to drop out^6,10^. Given the unpleasant consequences of burnout, it seems necessary to identify and analyze the contributing factors to prevent and eliminate it.

Previous studies have identified factors such as high academic workload^6,11^, social support^6,12–14^, institution support^13^, self-efficacy^6,15,16^, quality of learning experiences^15^, poor relationships with family, colleagues, and teachers^17^, lack of sense of community and social isolation^16^ as factors affecting students' academic burnout. Maslach and Leiter (1997) mentioned the factors affecting burnout as workload, lack of control, lack of rewards, lack of community, conflict of values, and lack of fairness^11^.

The high level of prevalence and severity of academic burnout among online students during the Covid-19 pandemic has been reported by numerous studies^13,18–21^. Pavlakis and Kaitelidou (2012) showed that distance learning can lead to problems such as depression, stress and burnout^22^. Yang, Chen & Chen (2021) highlighted that student workload, separation from faculty, and fear of contagion had negative effects on students' stress and health during Covid-19 pandemic^23^. Li, Fu, Fan, Zhu & Li (2021) in their study on the impact of the Covid-19 pandemic found that post-traumatic stress disorder due to various risk factors such as being infected to Covid-19, the problems of online classes such as little interaction with professors and classmates, disturbed learning environment and difficulty in adapting to these courses are common among students^24^. Brooks et al. (2020) in examining the psychological effects of quarantine, found that more than ten days of quarantine is associated with the possibility of post-traumatic stress symptoms^25^. One of the most important educational challenges for students during the Covid-19 pandemic is the pressure of homework and student duties^26,27^, especially the increase in workload in the online environment^27^.

Previous studies have pointed to problems and challenges such as the weakness of the online teaching infrastructure and platforms^28,29^, inexperienced teachers in online teaching^28,29^, the digital divide and the poor quality of Internet coverage, and the inadequacy of the home environment^29^. Naji, Do, Tarlochan, Ebead, Hasan, & Al-Ali (2020) identified initial preparedness and motivation for online learning, online learning self-efficacy beliefs, self-regulatory learning, and support as four important factors in preparing for online education^30^. Al-Adwan, Albelbisi, Hujran, Al-Rahmi, & Alkhalifah (2021) found that students’ satisfaction is affected by factors such as instructor, technical system, support service, educational systems, and course content quality^31^. Alkhawaja, Abd Halim, Abumandi & Al-Adwan (2022) found that system quality indirectly influences students actual use of online learning^32^.

Various studies have been conducted on student burnout and the factors affecting it in the face-to-face education, and less attention has been paid to the factors affecting burnout in the online environment, which are of a relatively different nature. Although after the end of Covid-19, most educational institutions have returned to the traditional face-to-face style, but many of these institutions have continued to implement online education in the post-Covid-19 era by gaining valuable experience during the coronavirus pandemic. According to the above, this study seeks to answer the following questions: what are the factors affecting students' academic burnout in online environments in the era of Covid-19? And how are the causal relationships between these factors? Knowing the factors affecting academic burnout and the causal relationships between them will help policymakers and planners to focus on the most effective factors, take more efficient and effective steps in better implementation of online education, and take more effective measures to reduce student burnout in the face of possible similar crises caused by pandemic conditions. This study can be different from previous studies in three ways. First, a comprehensive approach has been adopted in identifying the factors affecting academic burnout, while previous studies have been limited to two or three factors. Second, it focuses on academic burnout in online environments, and thirdly, in addition to identifying the effective factors, it examines the causal relationships between them in order to provide a model for decision making.

## Theoretical foundation

One of the theories that can be used to explain burnout and stress in the online learning environment is the resource conservation theory. According to this theory, people seek to acquire and maintain valuable resources^33,34^. The resource conservation theory implies that in situations where people face a high workload and excessive demand and perceive that the resources needed to do their job are threatened, or are not available, their perception of resource loss can lead to stress and burnout. Hobfoll (2018) categorizes the types of resources into physical and objective resources (such as valuable tools, physical facilities, and technology), personal resources (such as key skills, self-efficacy beliefs), condition resources (such as employment, interpersonal relationships, social support), and energy, resources that lead to access to other resources. (Such as knowledge and skills, time, and money)^34^.

The Job-Resource Demand (JD-R) model also highlights that the imbalance between resources needed to meet demands and available resources exposes people to stress^35^ and can lead to burnout. Studies on online education during the COVID-19 pandemic outbreak have also pointed to the provision of resources and support services to reduce pressure on students, increase their satisfaction and learning. Factors such as accessibility of resources, staff and student readiness, self-efficacy beliefs, access to new technologies and tools^36^, quality of course design and course delivery^37^, quality of student–teacher, student–student interactions, and Student-Content^38^, the speed and quality of the Internet network, the comfort and quietness of the learning environment, teacher support, and the ease of use of the learning platform (B and company 2020) have been emphasized^28,37^. Al-Kumaim, Alhazmi, Mohammed, Gazem, Shabbir, & Fazea (2021) in their integrated motivational model, proposed three categories of factors for sustainable well-being and mental health of students in online learning^39^. The factors included (1) individual factors (self-efficacy, self-regulation, and self-reliance including autonomy, competence, and connectivity), (2) technical factors (digital literacy, Attractive design, and mobile interactive learning), and (3) socio-environmental factors (family support, emotional engagement, university support including skilled educators, participatory culture, counseling support services).

## Method

In this study, the Gray Delphi method^40^ and, the Gray DEMATEL technique^41,42^ was used to identify the factors affecting student academic burnout. Matlab version R2020 software and Excel version 2021 software were employed for data analysis. DEMATEL is used for deriving the interrelationships between criteria. A grey DEMATEL method for group decision-making and analysis of the cause-effect relationship in grey environments was developed. The grey DEMATEL is applied to discriminate the cause-and-effect relationships of criteria. This help decision-makers to take more efficient actions by focusing on the ones with the greatest influence. The participants of the study were selected by one-stage cluster sampling method. For this purpose, out of 12 faculties, four faculties, and four departments from each faculty were randomly selected. In the Delphi stage, 86 graduate students and in the DEMATEL stage, 37 students (with the probability drop rate) were randomly selected from each department.

### Identification of factors

To explore factors affecting student academic burnout, an open questionnaire was administered to a randomly selected sample of 86 graduate students (Master courses and, Ph.D. courses) studying at Islamic Azad University, Science and Research Branch, and 43 factors were extracted.

### Gray Delphi method (GDM)

Dalkey and Helmer established Delphi method in 1963^43^. The Delphi method is a structured process for screening and ranking factors, which is implemented by gathering decision-makers' opinions through questionnaires. Three basic characteristics of the Delphi method are anonymous response, iteration and controlled feedback, and statistical group response^44,45^. Although the traditional Delphi method has been used in many studies, however, it has been criticized for its lengthy process, low convergence, and loss of some valuable expert information^46^. In addition, the traditional process of quantifying people's perspectives does not fully reflect the human thinking style due to the fuzziness, imprecision, and low compatible with linguistic and sometimes ambiguous human explanations, judgment, and priorities^45,47^. Grey system theory was initiated by^48^. The goal of the Grey System is to bridge the gap existing between social science and natural science^48^. In the grey system, all messages can be divided into three categories: white, grey, and black. The white part shows clear messages in a system ultimately, the black part has unknown characteristics, and the grey part happens between and covers both known and unknown messages. This theory includes four parts^49,50^.

Simultaneous use of the gray theory and the Delphi method is a proposed solution^40,51^.

*Step 1*: The Delphi questionnaire was distributed among 86 graduate students and asked them to determine the importance of each Criteria using linguistic variables. Linguistic variables and their corresponding gray scales for the importance weight of criteria according to^40^ are shown in Table 1.

**Table 1:** Linguistic variables for the importance of criteria.

Linguistic variables	Corresponding greyscale
Very unimportant (VU)	[1,2.5]
Unimportant (UN)	[0.5,3.5]
Fair (F)	[1.5,4.5]
Important (IM)	[2.5,5.5]
Very important (VI)	[3.5,5]

*Step 2:* Based on the method proposed by^40^, j ($j = 1,...,5$) gray classes were considered and, the selection range of ith criteria i.e.,$\left[ {a_{i}^{1} ,b_{i}^{5} } \right]$ was divided into 5 Gy classes.

*Step 3:* Eqs. (1) and (2) show the half trapezoidal whitening weight function applied for j = 1 and 5.

1 $$f_{i}^{1} (x) = \left\{ {\begin{array}{*{20}c} {\begin{array}{*{20}c} 1 & {x \le a_{i}^{1} } \\ \end{array} } \\ {\begin{array}{*{20}c} {\frac{{b_{i}^{1} - x}}{{b_{i}^{1} - a_{i}^{1} }}} & {a_{i}^{1} < x \le b_{i}^{1} } \\ \end{array} } \\ {\begin{array}{*{20}c} 0 & {x > b_{i}^{1} } \\ \end{array} } \\ \end{array} } \right.$$

2 $$f_{i}^{5} (x) = \left\{ {\begin{array}{*{20}c} {\begin{array}{*{20}c} 0 & {x \le a_{i}^{5} } \\ \end{array} } \\ {\begin{array}{*{20}c} {\frac{{x - a_{i}^{5} }}{{b_{i}^{5} - a_{i}^{5} }}} & {a_{i}^{5} \le x < b_{i}^{5} } \\ \end{array} } \\ {\begin{array}{*{20}c} 1 & {x \ge b_{i}^{5} } \\ \end{array} } \\ \end{array} } \right.$$

For J = 2,3,4. triangular whitening weight function as Eqs. (3) was applied.

3 $$f_{i}^{j} (x) = \left\{ {\begin{array}{*{20}c} {\begin{array}{*{20}c} 0 & {x \notin \left[ {a_{i}^{j} ,b_{i}^{j} } \right]} \\ \end{array} } \\ {\begin{array}{*{20}c} {\frac{{2(x - a_{i}^{j} )}}{{b_{i}^{j} - a_{i}^{j} }}} & {x \in \left[ {a_{i}^{j} ,\frac{{a_{i}^{j} + b_{i}^{j} }}{2}} \right]} \\ \end{array} } \\ {\begin{array}{*{20}c} {\frac{{2(b_{i}^{j} - x)}}{{b_{i}^{j} - a_{i}^{j} }}} & {x \notin \left[ {\frac{{a_{i}^{j} + b_{i}^{j} }}{2},b_{i}^{j} } \right]} \\ \end{array} } \\ \end{array} } \right.$$

*Step 4:* Eq. (4) was employed to calculate the synthetic clustering coefficient ($\rho_{i}^{j}$).

4 $$\rho_{i}^{j} = \sum\limits_{k = 1}^{m} {f_{i}^{j} (x) \cdot \eta_{i}^{k} }$$

*Step 5:* where $f_{i}^{j} (x)$ is the whitening weight function of jth’s grey class criteria i; m is the number of categories of students’ opinions; $\eta_{i}^{k}$ is the weight of criteria i in the synthetic cluster.

*Step 6:* The decision vectors of evaluation criteria were identified. The criterion $\max_{1 \le j \le 5} (\rho_{i}^{j} ) = \rho_{i}^{{j^{*} }}$ was used to judge whether criterion j belongs to the class $j^{*}$.

The selection criteria are as follows:

1. If class $j^{*}$ belong to classes 4 and 5, namely, the value of classes of important or very important is the maximum in the vector decision, the criterion is accepted.
2. If the ratio of class $j^{*}$ attached to class 4 and 5 to class $j^{*}$ attached to class 1 and 2 is more than 1, namely, classes of important and very important account for an over 50 percent degree except for the class of undecided, the criterion is accepted.

### Gray DEMATEL

Fontela used decision-Making Trial and Evaluation Laboratory (DEMATEL) technique at first and, Gabus in 1976^50^. DEMATEL is helpful in analyzing the cause-and-effect relationships among the components of a system. DEMATEL can demonstrate the existence of a relationship/interdependence among criteria or explain the relative level of relationships within them^50^. The DEMATEL does not depend on a large data sample and simplifies the correlation analysis of factors^52–54^. However, the traditional DEMATEL doesn't consider the fuzziness and uncertainties in the real-life^54–56^. Tseng (2009) expanded fuzzy triangular numbers to establish hierarchical grey DEMATEL to analyze criteria and alternatives in incomplete information^41^.

So, in this study, the gray DEMATEL technique was applied to develop the causal relationships model of factors affecting student academic burnout.

Step 1: A questionnaire with two square matrices (a square matrix of order 7 for the main criteria and, seven square matrices of order n, where n is a number of sub-criteria for each main criteria) was designed for pairwise comparison of the Criteria.

*Step 2:* A randomly selected sample of 37 graduate students (Master courses and, Ph.D. courses) evaluated interrelations among the Criteria by pairwise comparisons.

*Step 3:* The students used ten linguistic variables to illustrate the degree of causality between the Criteria. Linguistic variables and their corresponding gray-fuzzy numbers, according to^41,57,58^, to define the degree of influence of factors affecting student academic burnout are shown in Table 2.

**Table 2:** The linguistic Variables and their corresponding grey-fuzzy numbers.

Linguistic variables	Corresponding TFNs ($\otimes$ W)
Very low	(0.0, 0.3)
Low	(0.1, 0.5)
Medium	(0.3, 0.7)
High	(0.5, 0.9)
Very high	(0.7, 1.0)

*Step 4:* Assume $\otimes X$ an interval grey number is defined as $\otimes X = \left[ {\underline{X} ,\overline{X} } \right]$, and X's lower and upper bound is limited.

*Step 5:* Using Eq. (5) to aggregate students' opinions and a direct-relation matrix (n × n) ($i,j = 1, \ldots ,n$) was achieved to show that criteria i affects the criteria j.

5 $$\otimes X_{ij} = \frac{1}{h}\left( { \otimes X_{ij}^{1} + \otimes X_{ij}^{2} + \cdots + \otimes X_{ij}^{h} } \right)$$

6 $$X = \left[ {\begin{array}{*{20}c} { \otimes X_{11} } & \ldots & { \otimes X_{1n} } \\ \vdots & \vdots & \vdots \\ { \otimes X_{n1} } & \ldots & { \otimes X_{nn} } \\ \end{array} } \right]$$

*Step 6:* Normalized the grey relation decision matrix ($X^{\prime}$)

7 $$X{\prime} = \left[ {\begin{array}{*{20}c} { \otimes X_{11}{\prime} } & \ldots & { \otimes X_{1n}{\prime} } \\ \vdots & \vdots & \vdots \\ { \otimes X_{n1}{\prime} } & \ldots & { \otimes X_{nn}{\prime} } \\ \end{array} } \right]$$

*Step 7:* The relation normalized grey-DEMATEL decision matrix (M*).

8 $$M^{*} = \left[ {\begin{array}{*{20}c} { \otimes M_{11} } & \ldots & { \otimes M_{1n} } \\ \vdots & \vdots & \vdots \\ { \otimes M_{n1} } & \ldots & { \otimes M_{nn} } \\ \end{array} } \right]$$

Where

9 $$\otimes M_{ij} = \frac{{ \otimes X_{ij}{\prime} }}{{\max_{1 \le i \le n} \sum\limits_{j = 1}^{n} {M_{ij} } }}$$

*Step 8:* The total relation matrix (T)

10 $$T = M^{*} (I - M^{*} )^{ - 1}$$

where matrix I is the identity matrix of order n.

for transforming the grey weights into the crisp weights applies the average method, which is a simple and practical method to calculate the best non-grey performance (BNP) value of the grey weights of each aspect.

*Step 9:* The sum of each row and column of the total direct-relation matrix was stamped as two vectors $\overrightarrow{D}={\left[{d}_{i}\right]}_{n\times 1}$, $\vec{R} = \mathop {\left[ {r_{j} } \right]_{1 \times n} }\limits^{\prime }$, $\overrightarrow{D}$ + $\overrightarrow{R}$ and, $\overrightarrow{D}$—$\overrightarrow{R}$ vectors. $When i=j , if {d}_{i}>{r}_{j}\to {d}_{i}-{r}_{j}>0$, then the criterion is a net cause; $When i=j , if {d}_{i}<{r}_{j}\to {d}_{i}-{r}_{j}<0$, then criterion is a net effect. ${d}_{i}$ indicates the sum of direct and indirect effects of criterion i on other criteria. ${r}_{j}$ indicates the sum of direct and indirect effects on criterion j.

*Step 10:* A Cartesian coordinate system consisting of a horizontal axis ($\overrightarrow{D}$+$\overrightarrow{R}$) and a vertical axis ($\overrightarrow{D}$ -$\overrightarrow{R}$) was drawn in which the coordinates of each criterion are displayed in ordered pairs (${d}_{i}+{r}_{j}$ ,${d}_{i}-{r}_{j}$).

*Step 11:* Also determine the influential weights of criteria. The relative importance of the criteria is calculated by using the following equation**.**

11 $$W_{i} = \left[ {\left( {d_{i} + r_{i} } \right)^{2} + \left( {d_{i} - r_{i} } \right)^{2} } \right]^{\frac{1}{2}} \,\,\,\,\,\,\,\,\begin{array}{*{20}c} {\forall i} & {i = 1, \ldots ,n} \\ \end{array}$$

The normalized weight of any criterion was measured as follows:

12 $$\overline{W}_{i} = \frac{{W_{i} }}{{\sum\limits_{i = 1}^{n} {W_{i} } }}\,\,\,\,\,\,\,\,\,\begin{array}{*{20}c} {\forall i} & {i = 1, \ldots ,n} \\ \end{array}$$

where $\overline{W}_{i}$ shows the total criteria weights that would be required in the decision-making process. Therefore, therefore, the influential weight for each criterion (i.e., global influential weight) by applying the modified 2-tuple DEMATEL approach was calculated.

## Results

### Screening of factors affecting student academic burnout

The Gray Delphi represents identified factors affecting student academic burnout that are more important to the student. 86 students commented on the questionnaire with 43 criteria (factors affecting student academic burnout), divided into seven categories (see Table 3).

**Table 3:** Gray Delphi Method (GDM) Results.

Main factors	Sub-factors	Gray weight	Result
Students’ characteristics	Weakness in time management skills	(1.33, 2.67, 6.33, 6.67, 4.67)	Selected
	Incompatibility of learning and study style with online learning	(2.67, 5.33, 7.67, 3.67, 0.67)	Selected
	Lack of independence in learning	(3.67, 6.33, 6.00, 3.67, 1.33)	Selected
	Low technology self-efficacy	(1.33, 3.67, 6.33, 6.33, 2.67)	Selected
	Lack of online learning experience	(5.67, 6.33, 5.33, 2.33, 1.33)	Selected
	Weakness in skills of using new technologies	(4.33 7.67, 6.33, 2.67, 0.67)	Selected
	Lack of interest in online activities	(2.33, 3.67, 5.33, 5.33, 3.67)	Selected
Social/Emotional	sense of isolation due to physical distance in the online learning environment	(2.67, 5.33, 7.67, 3.67, 0.67)	Selected
	Lack of social cohesion and a sense of belonging in the online environment	(1.33, 3.67, 7.33, 5.67, 2.33)	Selected
	Little interactions between professors and students	(2.33, 3.67, 6.67, 6.67, 3.33)	Selected
Family/Home Environment	Imbalance between work, life and study	(4.33, 5.00, 6.333, 3.33, 0.67)	Selected
	unreasonable expectations of significant others	(1.33, 4.67, 9.00, 4.67, 1.67)	Rejected
	The difficulty of adapting to home environment in an online class	(3.67, 5.33, 6.33, 5.33, 2.67)	Selected
	Poor family relationships	(2.33, 6.67, 5.67, 5.33, 1.33)	Rejected
	Family problems	(3.33, 5.67, 6.00, 5.67, 1.67)	Rejected
	excessive family and work duties and responsibilities	(4.33, 5.00, 6.333, 3.33, 0.67)	Selected
	Lack of sufficient space at home to study and learn	(2.67, 2.67, 6.33, 6.33, 5.67)	Selected
	Lack of comfort and silence at home to learn	(2.33, 5.00, 5.67, 5.33, 1.33)	Rejected
	Concerns about getting the Corona virus	(2.33, 4.67, 5.33, 7.00, 3.67)	Selected
Technology infrastructure	Poor quality and lack of user-friendly system and online learning portal	(3.33, 5.67, 5.33, 4.33, 2.00)	Selected
	Impossibility of using multimedia in the designed online learning environment	(3.67, 6.33, 7.33, 3.67, 0.67)	Selected
	Low speed and poor quality of internet and communication breakdowns	(2.67, 3.33, 5.00, 5.67, 3.33)	Selected
	Digital divide (unequal access to quality internet at a reasonable price	(2.33, 5.67, 7.00, 4.00, 1.33)	Rejected
	The Difficulty in working with the designed learning environment system	(5.67, 6.33, 5.33, 2.67, 0.67)	Selected
	The Difficulty in accessing hardware equipment due to high cost	(4.33, 6.67, 7.00, 3.33, 0.67)	Selected
	Lack of resources and tools for interactive learning	(2.67, 4.33, 5.67, 7.67, 3.33)	Selected
Institutional facilities	Weakness of regular technical support system	(4.33, 6.67, 7.00, 3.33, 0.67)	Selected
	poor online services	(0.67, 2.33, 5.67, 9.33, 4.33)	Selected
	Lack of a proper and convenient communication system between the university and students	(7.67, 6.33, 3.33, 2.67, 1.33)	Selected
	Lack of timely and appropriate online response to the students	(3.67, 5.33, 5.67, 4.33, 2.33)	Selected
Educational	Limited class time to resolve students' problems and ambiguities	(6.33, 7.33, 3.67, 2.00, 0.67)	Selected
	Insufficient training on using online space for students and professors	(2.67, 3.33, 5.67, 6.33, 3.33)	Selected
	Improper design of courses to fit the online space	(1.33, 2.67, 5.33, 8.67, 4.67)	Selected
	Lack of interactive online classroom environment compared to face-to-face training	(1.33, 2.67, 6.00, 9.33, 5.67)	Selected
	Limited participation of students in class activities for technical and technological reasons	(2.67, 4.33, 5.67, 7.67, 3.33)	Selected
	Lack of variety of tools and methods of learning and teaching in the online learning	(5.67, 6.33, 5.33, 2.33, 1.33)	Selected
	Distraction and low concentration of students in the online learning environment	(3.67, 3.33, 6.33, 5.33, 1.33)	Selected
Teachers’ characteristics	Teachers’ unavailability for guidance and students supports outside the classroom	(2.67, 3.67, 5.00, 5.67, 3.33)	Selected
	Negative attitude of teachers to online teaching and learning	(3.33, 1.67, 3.67, 8.33, 4.67)	Selected
	poor skills of teachers in the optimal use of online space	(6.33, 2.67, 5.33, 9.33, 3.67)	Selected
	Low experience of professors in using new technologies	(1.67,5.33, 7.33, 4.00, 0.67)	Rejected
	Limited experience of teachers in applying new technologies	(1.33, 2.67, 6.33, 6.67, 4.67)	Selected
	Low self-efficacy of teachers in the effective use of online space	(6.33, 7.67, 3.67, 2.67, 2.67)	Selected

According to the students' opinion, out of the 43 factors identified, six sub-factors were less important in their academic burnout, so these six sub-factors were removed from the list of influential factors, and 37 more important factors were used to determine the model.

### Gray DEMATEL calculations and results

**Figure 1:**
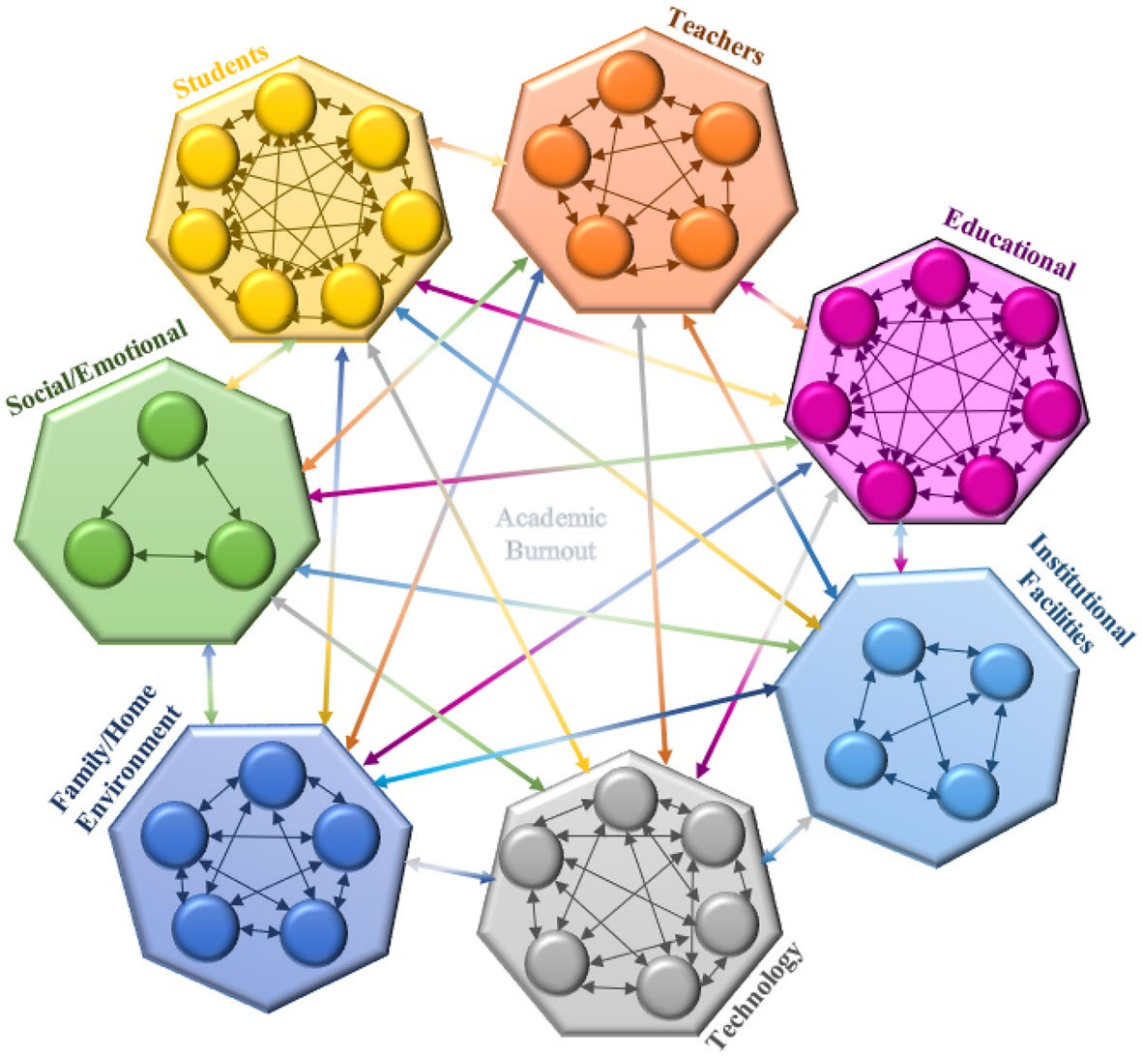
Initial diagram of factors affecting Student academic burnout.

After determining the most important factors affecting student academic burnout, the corresponding initial diagram was drawn so that its vertices are the same as the components of the system and its arc is the degrees of direct relations between both criteria of the system and the intensity of the effect of each direct relation on the corresponding are is showing (see Fig. 1).

The $\overrightarrow{D}$, $\overrightarrow{R}$, $\overrightarrow{D}$ + $\overrightarrow{R}$ and, $\overrightarrow{D}$ − $\overrightarrow{R}$ vectors for showing the “Prominence” and “Relation” values for main factors and sub-factors base on gray DEMATEL were calculated, where can be seen in Tables 4, 5, 6, 7, 8, 9, 10 and 11.

**Table 4:** The “Prominence” and “Relation” values for main factors.

Main criteria	Influencing factors	Influenced factors	Interrelationship	Net cause/Net effect	Weight	Normal weight
	$\overrightarrow{D}$	$\overrightarrow{R}$	$\overrightarrow{D}$+$\overrightarrow{R}$	$\overrightarrow{D}$− $\overrightarrow{R}$	$W_{i}$	$\overline{W}_{i}$
Students’ characteristics	0.407	1.047	1.454	− 0.64	1.589	0.135
Social/Emotional	0.237	1.195	1.433	− 0.958	1.723	0.146
Family/Home Environment	0.454	0.613	1.067	− 0.158	1.078	0.092
Technology infrastructure	1.627	0.093	1.72	1.534	2.304	0.196
institutional facilities	1.101	0.237	1.339	0.864	1.593	0.135
Educational	0.597	1.404	2.001	− 0.807	2.158	0.183
Teachers’ characteristics	0.738	0.572	1.309	0.166	1.320	0.112

**Table 5:** The “Prominence” and “Relation” values for sub-factors of student characteristics.

Students’ characteristics	Influencing factors	Influenced factors	Interrelationship	Net cause/Net effect	Weight	Normal weight
	$\overrightarrow{D}$	$\overrightarrow{R}$	$\overrightarrow{D}$+$\overrightarrow{R}$	$\overrightarrow{D}$ − $\overrightarrow{R}$	$W_{i}$	$\overline{W}_{i}$
Weakness in time management skills	0.660	0.082	0.742	0.577	0.940	0.071
Incompatibility of learning and study style with online learning	0.517	0.120	0.637	0.396	0.750	0.057
Lack of independence in learning	0.632	0.838	1.469	− 0.206	1.484	0.112
Low technology self-efficacy	1.034	0.901	1.935	0.133	1.939	0.147
Lack of online learning experience	1.140	1.251	2.391	− 0.112	2.394	0.181
Weakness in skills of using new technologies	1.489	1.283	2.772	0.206	2.779	0.211
Lack of interest in online activities	0.871	1.866	2.737	− 0.995	2.913	0.221

**Table 6:** The “Prominence” and “Relation” values for sub-factors of Social/Emotional.

Social/Emotional	Influencing factors	Influenced factors	Interrelationship	Net cause/Net effect	Weight	Normal weight
	$\overrightarrow{D}$	$\overrightarrow{R}$	$\overrightarrow{D}$ + $\overrightarrow{R}$	$\overrightarrow{D}$ − $\overrightarrow{R}$	$W_{i}$	$\overline{W}_{i}$
sense of isolation due to physical distance in the online learning environment	0.606	1.183	1.789	− 0.578	0.940	0.296
Lack of social cohesion and a sense of belonging in the online environment	0.606	1.183	1.789	− 0.578	0.750	0.236
Little interactions between professors and students	1.606	0.450	2.056	1.156	1.484	0.467

**Table 7:** The “Prominence” and “Relation” values for sub-factors of Family/Home Environment.

Family/Home environment	Influencing factors	Influenced factors	Interrelationship	Net cause/Net effect	Weight	Normal weight
	$\overrightarrow{D}$	$\overrightarrow{R}$	$\overrightarrow{D}$ + $\overrightarrow{R}$	$\overrightarrow{D}$ − $\overrightarrow{R}$	$W_{i}$	$\overline{W}_{i}$
Imbalance between work, life and study	0.609	1.745	2.353	− 1.136	2.613	0.252
The difficulty of adapting to home environment in an online class	0.883	1.456	2.339	− 0.573	2.408	0.232
excessive family and work duties and responsibilities	1.315	0.955	2.270	0.359	2.298	0.221
Lack of sufficient space at home to study and learn	1.474	0.669	2.142	0.805	2.289	0.221
Concerns about getting the Coronavirus	0.545	0.000	0.545	0.545	0.771	0.074

**Table 8:** The “Prominence” and “Relation” values for sub-factors of Technology.

Technology	Influencing factors	Influenced factors	Interrelationship	Net cause/Net effect	Weight	Normal weight
	$\overrightarrow{D}$	$\overrightarrow{R}$	$\overrightarrow{D}$ + $\overrightarrow{R}$	$\overrightarrow{D}$− $\overrightarrow{R}$	$W_{i}$	$\overline{W}_{i}$
Poor quality and lack of user-friendly system and online learning portal	0.922	0.535	1.457	0.387	1.508	0.168
Impossibility of using multimedia in the designed online learning environment	0.417	1.424	1.841	− 1.007	2.099	0.234
Low speed and poor quality of internet and communication breakdowns	0.941	0.000	0.941	0.941	1.330	0.148
The Difficulty in working with the designed learning environment system	0.560	1.494	2.054	− 0.933	2.257	0.252
The Difficulty in accessing hardware equipment due to high cost	0.548	0.120	0.668	0.428	0.794	0.089
Lack of resources and tools for interactive learning	0.572	0.388	0.960	0.184	0.978	0.109

**Table 9:** The “Prominence” and “Relation” values for sub-factors of Institutional facilities.

Institutional facilities	Influencing factors	Influenced factors	Interrelationship	Net cause/Net effect	Weight	Normal weight
	$\overrightarrow{D}$	$\overrightarrow{R}$	$\overrightarrow{D}$+$\overrightarrow{R}$	$\overrightarrow{D}$ − $\overrightarrow{R}$	$W_{i}$	$\overline{W}_{i}$
Weakness of regular technical support system	0.784	0.169	0.953	0.614	1.134	0.219
poor online services	0.559	0.449	1.008	0.110	1.014	0.196
Lack of a proper and convenient communication system between the university and students	0.811	0.247	1.058	0.564	1.199	0.232
Lack of timely and appropriate online response to the students	0.000	1.288	1.288	− 1.288	1.822	0.353

**Table 10:** The “Prominence” and “Relation” values for sub-factors of educational factor.

Educational	Influencing factors	Influenced factors	Interrelationship	Net cause/Net effect	Weight	Normal weight
	$\overrightarrow{D}$	$\overrightarrow{R}$	$\overrightarrow{D}$+$\overrightarrow{R}$	$\overrightarrow{D}$ − $\overrightarrow{R}$	$W_{i}$	$\overline{W}_{i}$
Limited class time to resolve students' problems and ambiguities	0.529	0.586	1.115	− 0.057	1.117	0.110
Insufficient training on using online space for students and professors	1.555	0.079	1.635	1.476	2.202	0.217
Improper design of courses to fit the online space	0.820	0.417	1.237	0.404	1.301	0.128
Lack of interactive online classroom environment compared to face-to-face training	0.306	1.256	1.562	− 0.951	1.829	0.180
Limited participation of students in-class activities for technical and technological reasons	0.612	0.259	0.870	0.353	0.939	0.092
Lack of variety of tools and methods of learning and teaching in the online learning	0.474	0.428	0.902	0.046	0.903	0.089
Distraction and low concentration of students in the online learning environment	0.058	1.328	1.386	− 1.271	1.880	0.185

**Table 11:** The "Prominence" and "Relation" values for sub-factors of Teachers' characteristics.

Educational	Influencing factors	Influenced factors	Interrelationship	Net cause/Net effect	Weight	Normal weight
	$\overrightarrow{D}$	$\overrightarrow{R}$	$\overrightarrow{D}$+$\overrightarrow{R}$	$\overrightarrow{D}$ − $\overrightarrow{R}$	$W_{i}$	$\overline{W}_{i}$
Teachers' unavailability for guidance and student supports outside the classroom	0.000	1.100	1.100	− 1.100	1.556	0.133
The negative attitude of teachers to online teaching and learning	1.683	1.054	2.738	0.629	2.809	0.239
poor skills of teachers in the optimal use of online space	1.575	0.754	2.328	0.821	2.469	0.210
Limited experience of teachers in applying new technologies	1.862	0.754	2.616	1.109	2.841	0.242
Low self-efficacy of teachers in the effective use of online space	0.000	1.458	1.458	− 1.458	2.062	0.176

The most influencing factors are technology infrastructures, Academic/Institutional facilities and Teacher’s characteristics, respectively. The most influenced factors are educational, social/emotional and student characteristics factors, respectively. The most interrelationship factors (total influencing and influenced factors) are educational/classroom environment and lesson structure, technology infrastructures and individual factors (student characteristics), respectively.

The highest influencing factors among students’ characteristics are weakness in skills of using new technologies, lack of online learning experience and low technology self-efficacy (lack of believing incapability of using technologies in learning), respectively. The highest influenced factors are lack of interest in online activities, weakness in skills of using new technologies, and lack of online learning experience, respectively. The most interrelationship factors (total influencing and influenced factors) are weakness in skills of using new technologies, lack of interest in online activities, and lack of online learning experience, respectively. The factors of weakness in time management skills, incompatibility of learning and study style with online learning, low technology self-efficacy (lack of believing incapability of using technologies in learning), and weakness in skills of using new technologies are net causes, and lack of independence in learning, lack of online learning experience and lack of interest in online activities are net effects.

Among social/emotional factors, the most influencing factor is little interactions between professors and students. The most influenced factors are sense of isolation due to physical distance in the online learning environment and lack of social cohesion and a sense of belonging in the online environment. The most interrelationship factor (total influencing and influenced factors) is little interaction between professors and students. Little interactions between professors and students are net cause and sense of isolation due to physical distance in the online learning environment and lack of social cohesion and a sense of belonging in the online environment are net effects.

Among factors related to family/home environment, the highest influencing factors are lack of sufficient space at home to study and learn, excessive family and work duties and responsibilities, and the difficulty of adapting to the home environment in an online class, respectively. The most influenced factors are the imbalance between work, life and study, the difficulty of adapting to the home environment in an online class, and excessive family and work duties and responsibilities. The most interrelationship factors (total influencing and influenced factors) are imbalance between work, life and study, the difficulty of adapting to home environment in an online class and excessive family and work duties and responsibilities, respectively. The factors of lack of sufficient space at home to study and learn, concerns about getting the coronavirus and excessive family and work duties and responsibilities are net causes and imbalance between work, life and study, and the difficulty of adapting to home environment online classes are net effects.

Among factors related to technology and infrastructures, the most influencing factors are low speed and poor quality of internet and communication breakdowns and poor quality and lack of user-friendly system and online learning portal, respectively. The most influenced factors are the difficulty in working with the designed learning environment system and the impossibility of using multimedia in the designed online learning environment, respectively. The low speed and poor quality of internet and communication breakdowns have no effect. The most interrelationship factors (total influencing and influenced factors) are the difficulty in working with the designed learning environment system, impossibility of using multimedia in the designed online learning environment, and poor quality and lack of user-friendly system and online learning portal respectively. The factors of low speed and poor quality of internet and communication breakdowns, the difficulty in accessing hardware equipment due to high cost, poor quality and lack of user-friendly system and online learning portal and lack of resources and tools for interactive learning are net causes and impossibility of using multimedia in the designed online learning environment, and the difficulty in working with the designed learning environment system is net effects.

Among Institutional facilities and infrastructures, the most influencing factors are the lack of a proper and convenient communication system between the university and students and the weakness of the regular technical support system. The lack of timely and appropriate online response to the students has no effect. The most influenced factors are lack of timely and appropriate online response to the students by the university, poor online services and lack of a proper and convenient communication system between the university and students, respectively. The most interrelationship factors (total influencing and influenced factors) are lack of timely and appropriate online response to the students through online space by the university and lack of a proper and convenient communication system between the university and students, respectively. The factors of the weakness of regular technical support system, lack of a proper and convenient communication system between the university and students, poor online services are net causes, and lack of timely and appropriate online response to the students is net effect.

Among educational factors/classroom environment and lesson structure, the most influencing factors are insufficient training on using online space for students and professors, improper design of courses to fit the online space, limited participation of students in-class activities for technical and technological reasons, and limited class time to resolve students' problems and ambiguities, respectively. The most influenced factors are distraction and low concentration of students in the online learning environment, lack of interactive online classroom environment compared to face-to-face training, and limited class time to resolve students' problems and ambiguities, respectively. The most interrelationship factors (total influencing and influenced factors) are insufficient training on using online space for students and professors, lack of interactive online classroom environment compared to face-to-face training and distraction, and low concentration of students in the online learning environment, respectively. The factors of insufficient training on using online space for students and professors, improper design of courses to fit the online space and limited participation of students in-class activities for technical and technological reasons are net causes and distraction and low concentration of students in the online learning environment, lack of interactive online classroom environment compared to face-to-face training, limited class time to resolve students' problems and ambiguities are net effects .

Among factors related to teachers, the most influencing factors are low self-efficacy of teachers in the effective use of online space, negative attitude of teachers to online teaching and learning and poor skills of teachers in the optimal use of online space, respectively. The factors of teachers' unavailability for guidance and student supports outside the classroom and low self-efficacy of teachers in the effective use of online space have no effects. The most influenced factors are low self-efficacy of teachers in the effective use of online space, teachers’ unavailability for guidance and students supports outside the classroom and negative attitude of teachers to online teaching and learning, respectively. The most interrelationship factors (total influencing and influenced factors) are the negative attitude of teachers to online teaching and learning, low self-efficacy of teachers in the effective use of online space and poor skills of teachers in the optimal use of online space, respectively. The factors of limited experience of teachers in applying new technologies, poor skills of teachers in the optimal use of online space and negative attitude of teachers to online teaching and learning are net causes and low self-efficacy of teachers in the effective use of online space and teachers’ unavailability for guidance and students supports outside the classroom are net effects. The relative importance of each main factor and subfactors is shown in the Table 12.

**Table 12:** Comparative weight of each factor.

Main Factors	Subfactors	Local normalized weight	Global normalized weight
Students’ characteristics (0.135)	Weakness in time management skills	0.071	0.010
	Incompatibility of learning and study style with online learning	0.057	0.008
	Lack of independence in learning	0.112	0.015
	Low technology self-efficacy (lack of believing incapability of using technologies in learning)	0.147	0.020
	Lack of online learning experience	0.181	0.024
	Weakness in skills of using new technologies	0.211	0.028
	Lack of interest in online activities	0.221	0.030
Social/Emotional (0.146)	sense of isolation due to physical distance in the online learning environment	0.296	0.043
	Lack of social cohesion and a sense of belonging in the online environment	0.236	0.035
	Little interactions between professors and students	0.467	0.068
Family/Home Environment (0.091)	Imbalance between work, life and study	0.252	0.023
	The difficulty of adapting to home environment in an online class	0.232	0.021
	excessive family and work duties and responsibilities	0.221	0.020
	Lack of sufficient space at home to study and learn	0.221	0.020
	Concerns about getting the Corona virus	0.074	0.007
Technology (0.196)	Poor quality and lack of user-friendly system and online learning portal	0.168	0.033
	Impossibility of using multimedia in the designed online learning environment	0.234	0.046
	Low speed and poor quality of internet and communication breakdowns	0.148	0.029
	The Difficulty in working with the designed learning environment system	0.252	0.049
	The Difficulty in accessing hardware equipment due to high cost	0.089	0.017
	Lack of resources and tools for interactive learning	0.109	0.021
Academic facilities (0.135)	Weakness of regular technical support system	0.219	0.030
	poor online services	0.196	0.027
	Lack of a proper and convenient communication system between the university and students	0.232	0.031
	Lack of timely and appropriate online response to the students	0.353	0.048
Educational (0.183)	Limited class time to resolve students' problems and ambiguities	0.110	0.020
	Insufficient training on using online space for students and professors	0.217	0.040
	Improper design of courses to fit the online space	0.128	0.023
	Lack of interactive online classroom environment compared to face-to-face training	0.180	0.033
	Limited participation of students in class activities for technical and technological reasons	0.092	0.017
	Lack of variety of tools and methods of learning and teaching in the online learning	0.089	0.016
	Distraction and low concentration of students in the online learning environment	0.185	0.034
Teacher’s characteristics (0.112)	Teachers’ unavailability for guidance and students supports outside the classroom	0.133	0.015
	Negative attitude of teachers to online teaching and learning	0.239	0.027
	poor skills of teachers in the optimal use of online space	0.210	0.024
	Limited experience of teachers in applying new technologies	0.242	0.027
	Low self-efficacy of teachers in the effective use of online space	0.176	0.020

**Figure 2:**
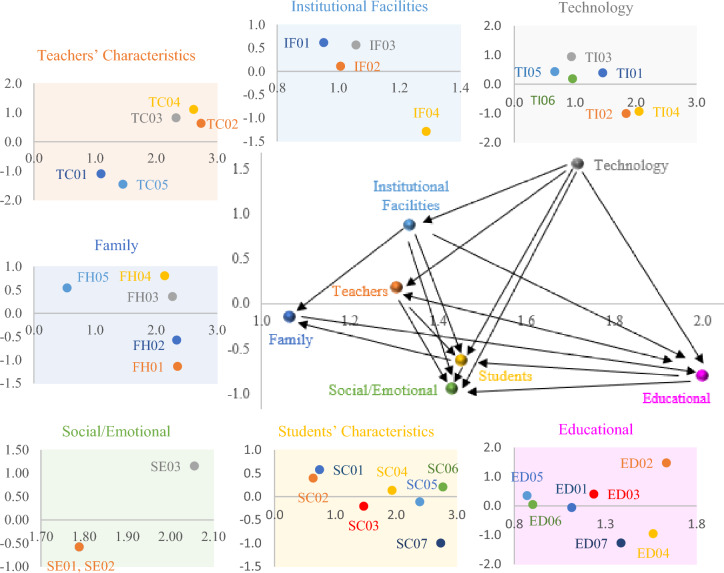
The impact relationship map for criteria and sub-criteria.

The order of main factors is Technology with the relative importance of 0.196, Educational with the relative importance of 0.183, Social/Emotional with the relative importance of 0.146, Individual and, Academic facilities with the relative importance of 0.135, Teachers’ characteristics with the relative importance of 0.112 and Family/Home Environment with the relative importance of 0.091, respectively. Among subfactors, little interactions between professors and students, the Difficulty in working with the designed learning environment system, lack of timely and appropriate online response to the students, impossibility of using multimedia in the designed online learning environment, sense of isolation due to physical distance in the online learning environment, insufficient training on using online space for students and professors, lack of social cohesion and a sense of belonging in the online environment, distraction and low concentration of students in the online learning environment, poor quality and lack of user-friendly system and online learning portal, lack of interactive online classroom environment compared to face-to-face training had the highest weight and concerns about getting the Coronavirus, incompatibility of learning and study style with online learning, weakness in time management skills, lack of independence in learning, teachers' unavailability for guidance and students supports outside the classroom had the lowest weights (see Fig. 2).

The factors of technology infrastructures, organizational/academic facilities, and infrastructure and factors related to teachers are included in net causes and the factors of individual factors (individual particulars), social/emotional and educational/classroom environment and lesson structure are included in net effects. The technology infrastructures and facilities have the highest influence and is the net cause, so it is a core factor. It should be priorities in terms of improvement and its improvement shall affect other factors and could solve a big part of issues. The organizational/academic facilities and infrastructure and factors related to teachers have high effectiveness but less than technology infrastructures and facilities and are net causes, hence they are driving factors, i.e., could be further priorities in terms of improvement and their improvement could relatively affect others’ improvement.

Educational/classroom environment and lesson structure factor is net effect and has high interrelationship, so this is impact factor which is affected by other factors and its improvement couldn’t such affect directly.

## Conclusion

Due to the outbreak of COVID-19 and the necessity of shift to online education, one of the concerns in educational settings had been the prevalence of burnout among students. Therefore, this study has sought 1) to identify the factors affecting students' academic burnout 2) to determine the causal relationships (interaction) of the identified factors. Findings on screening factors extracted from interviews with students showed that out of 43 extracted sub-factors, 37 sub-factors in 7 main factors (technology infrastructure, institution facilities, faculty characteristics, educational factors/classroom environment and course structure, Individual/student characteristics factor, Social-emotional factor, family and home environment Factor) affect students' academic burnout. One of the most important data analysis findings through DEMATEL is the division of identified factors into two groups: net cause and net effect groups. The findings of this study, consistent with the findings of previous studies^59,60^ show that academic burnout is not a syndrome that occurs only due to one or more limited and separate factors, but is the result of the interaction of several factors. The factors of the cause group, in order of magnitude of impact, are: technology infrastructure, institution facilities, and characteristics of the professors. The effect group are: educational factors/classroom environment and course structure, Individual/student characteristics factor, Social-emotional factor, family and home environment Factor, respectively. Of the seven factors, the most influential is the technology infrastructure factor which is called the core factor, and the two factors of institutional facilities and the characteristics of professors with less impact than the technology factor are identified as driving factors. The findings of this study showed that technology infrastructure is the most effective factor among the seven factors and has the greatest impact on other factors and academic burnout. The findings of this study about the factors affecting students' academic burnout and the causal relationships between them can be well explained by^33,34^ conservation of resource (COR) theory. According to COR theory, people try to obtain and retain resources (what is valuable to them) but losing resources is more important than gaining resources (Principle 1). Stress occurs when a key and important resource is lost or at risk of being lost^34^. When people do not have access to key resources (high-speed and quality Internet, regular support services, interactive learning tools, etc.) or conditions threaten their ability to obtain or retain important and key resources, they are exposed to stress and burnout.

As the results show about the technology factor according to the theory of resource conservation^33,34,61^, low speed and poor internet quality, and communication interruptions, poor quality and lack of user-friendly online learning system and portal, and lack of resources and interactive learning tools lead to the difficulty of working with the learning environment system and the impossibility of using multimedia in the online learning environment. It also threatens the student with the loss of other important resources, such as learning, gaining knowledge and skills, and engaging in educational activities. This acts as a stressor and lead students burn out. The finding that poor technology infrastructure is a major contributor to student burnout is consistent with the findings of^29^ who reported that the digital divide and poor quality of students' Internet networks pose a significant challenge to online education. Findings show that the factor of institutional facilities and organizational infrastructure is the second factor in terms of the impact on students' academic burnout. This finding is consistent with the job-resource demand model that the imbalance between demand and resources needed to meet requirements lead to burnout^35^. The theory of resource conservation^34^ also states that lack of resources (institutional facilities and support) can lead to loss of other resources (such as learning and development opportunities) and cause dissatisfaction and burnout in students. The causal relationship between the sub-factors shows that poor technical support system, lack of a proper communication system between the university and students, and insufficient online services (as the net cause sub-factors) lead to lack of timely and appropriate response to students (as the net effect sub-factor). This study, in line with the study of^6,12,13,28,37^ shows that support (either from professors or from the institution) is an important factor in student burnout. The third net influencing factor among the seven factors is the characteristics of the professors.

The role of teachers' beliefs, attitudes and skills as the main pillar of education in all teaching styles is undeniable. But in online education, the role of the teacher is more prominent and is a determining factor in helping students adapt to the online learning environment and reduce their stress and increase their mental health. The causal relationship between the sub-factors highlights that faculty members' negative attitudes, low self-efficacy and low faculty skills in the effective use of online space (as cause sub-factors) lead to their low control over the classroom, and their unavailability for guidance and support of students (as net effect sub-factors). This finding is consistent with the findings^28^ and^29^ study that poor online teaching infrastructure and teachers' lack of experience in online education are troublesome and stressful for students. As mentioned before, In the group of effect factors, the most influential factors are education/classroom environment and course structure (the most influential factor), social-emotional factor, students' personal characteristics, and home and family environment, respectively. The education factor (classroom environment and course structure) is directly influenced by the three factors of technology infrastructure, institutional facilities and support services, and the characteristics of the professors. Given the differences between face-to-face and online classes, for a smooth and stress-free transition to online education and reducing burnout, it is necessary to provide counseling services, train professors and students to work with online platforms, and design courses tailored to the online space. Also, the use of appropriate educational platforms and interactive tools can help increase classroom interactions, sense of community and reduce social isolation in students.

Causal relationships between social emotional factor sub-factors also show that low interactions between faculty and students, and students -students (net effect) lead to sense of isolation, lack of social cohesion and lack of sense of belonging in the online environment (net effect). This study confirms the findings of^16,24,25,62^, studies on the impact of low interactions and a sense of social isolation on students' mental health and burnout. Analysis of causal relationships between factors shows that the factor of student characteristics is the third factor that is affected by three factors: technology, facilities of the institution and the characteristics of professors. Based on causal relationships between student characteristics sub-factors, poor time management skills, inappropriate learning and study style, low technology self-efficacy and lack of mastery in using new technologies (net cause sub-factors) result in lack of learning autonomy, low online learning experience and lack of interest in online activities (net effect sub-factors). The effect of low technology and Internet self-efficacy of students on student burnout has also been mentioned in the findings of^6,15,16,30^. In this study, self-efficacy refers to students' beliefs and judgments about their ability to work with new technologies and online learning platforms that can be influenced by individual and environmental factors. Due to the interactions between the factors affecting burnout, this can be due to the unpreparedness of students and professors and lack of previous knowledge, skills and experience in working with new platforms. If this lack of knowledge, skills, and experience is not compensated by receiving adequate training, and regular and quality support services from the university, it can expose students to stress and burnout (principles of Resource Conservation Theory).

Findings show that the least influential factor among the seven factors is the home and family environment. Analysis of causal relationships of sub-factors highlights inappropriate of the home for study and learning, the stress and concern of being infected with the coronavirus, and heavy family and work responsibilities (net effect) lead to an imbalance between work, life and study, and the difficulty of adapting the home environment to online learning (net effect). Impact of fear of infection with the coronavirus on student burn out has been reported in^21^ study. This finding is consistent with the findings of^29^ that the inadequacy of the home environment in online education is a problem that leads to student stress. Generally, transition from one situation to another by taking people out of the comfort zone causes them stress and psychological unsafety and can lead to burnout. In other words, if the conditions and facilities (high-speed and quality Internet network, user-friendly online learning platforms, interactive tools, qualified professors), support services (regular online support services, continuous communication with students and answering their ambiguities and questions, faculty support Students and responding to them in a timely manner), and not making the necessary preparations (providing the necessary training to students and faculty members) will lead to burnout.

The findings of this study are consistent with the findings of^62^ on internal barriers (attitudes and beliefs, resistance to technology use, and knowledge and skills) and external barriers (access, training, and support) to technology use in the classroom, and it also confirms the findings of^30^ on the role of early preparation, self-efficacy beliefs, and support in transitioning to online education.

## Implication and contribution

Considering the possibility of continuing online education courses alongside face-to-face education in the post-corona era, identifying the affecting factors of students 'academic burnout in online education and finding the causal relationships between them (determining the cause group factors and the effect group factors) can be used as a shortcut to take smart measures to prevent and reduce students' burnout and make the learning environment enjoyable in implementing online education in the future and facing possible pandemic crises.

The most important theoretical contribution of this study is to develop a model based on causal relationships between the factors affecting burn out and dividing them into two categories: the cause group and the effect group. In the sense that it is not limited to identifying and screening the factors affecting academic burnout, instead, considering the interaction of factors and their inseparability through the gray DEMATEL technique. Finding the causal relationships between factors and determine the cause group and the effect group can play a catalytic role in the formulation and implementation of intervention strategies and programs in educational institutions.

The findings of this study suggest that to prevent and reduce student burnout in the online environment, seven effective factors should be considered, but in order to manage resources and increase the efficiency and effectiveness of measures in this field, a logical step is to focus on the three net cause factors of technology infrastructure, institutional facilities, The faculty members characteristics. By manipulating and investing in these three categories of factors, we are expected to see changes in the next four categories of factors (education/ class environment and course structure, social and emotional factor, student characteristics factor, and home and family factor).

The most important limitation of this study is that the model of effective factors and causal relationships between them was the result of analyzing the participants' perspective data in the paired matrix through the DEMATEL technique. Future researchers can provide fitted models for different settings by collecting real data and analyzing data using structural equation modeling techniques.

## Data Availability

All data generated or analyzed during this study are included in this published article.
